# An Unexpected Cause of Shock in a Trauma Patient with Hemodynamic Instability: A Case Report

**DOI:** 10.5811/cpcem.43502

**Published:** 2025-07-29

**Authors:** Natalie A. Jansen, Christie Fritz

**Affiliations:** Beth Israel Deaconess Medical Center, Department of Emergency Medicine, Boston, Massachusetts

**Keywords:** case report, eFAST, trauma, pulmonary embolism

## Abstract

**Introduction:**

Traumatic injury is the leading cause of death in individuals under 45 years of age, and point-of-care ultrasound (POCUS) has become an essential component of the initial trauma evaluation. However, positive findings on the extended focused assessment with sonography in trauma (eFAST) may be misinterpreted as evidence of an acute surgical emergency, particularly in the context of blunt trauma, underscoring the need for careful clinical correlation.

**Case Report:**

We present a case in which a hemodynamically unstable patient had significant free abdominal fluid on eFAST after a fall from standing height. She was ultimately diagnosed with a high-risk pulmonary embolism as the cause of her hemodynamic instability, while the free abdominal fluid was identified as originating both from a ruptured ovarian cyst and from moderate-volume ascites.

**Conclusion:**

The eFAST exam is a valuable tool in rapidly identifying intra-abdominal injuries following blunt trauma. However, the presence of free fluid on eFAST may result from causes other than acute intra-abdominal injury requiring surgical intervention. Therefore, emergency physicians should interpret positive findings with clinical judgment and consider the broader clinical context.

## INTRODUCTION

Traumatic injury is the leading cause of death in individuals under 45 years of age in the United States.^1^ Blunt abdominal injuries may cause significant bleeding and hemodynamic instability, and quickly identifying life-threatening injuries is a crucial component of the initial trauma evaluation. Adoption of point-of-care ultrasound on initial trauma evaluation has led to significant reductions in time to operative management and in overall medical costs.^2^ Extended focused assessment with sonography in trauma (eFAST) can helpfully rule in, but not rule out, free intra-abdominal fluid in hypotensive adults with a sensitivity of 74% and specificity of 95%.^3^,^4^ Fluid accumulation becomes visible in the hepatorenal or splenorenal spaces when it exceeds 500 milliliters (mL), whereas as little as 150 mL can be detected in the pelvic view.^5^ The presence of fluid without evidence of intra-abdominal injury may arise from causes such as ovarian cyst rupture (traumatic or atraumatic), ascites, peritoneal dialysate,^6^ or physiologic fluid (up to 50 mL)^5^ in women of childbearing age.^7^ In this case, we describe a high-risk pulmonary embolism (PE) in an otherwise healthy female who presented for trauma evaluation after a fall from standing height and was found to have a grossly positive eFAST exam.

## CASE REPORT

An 18-year-old female without significant past medical history presented as a transfer from an outside hospital with hypotension and a positive eFAST exam after a fall from standing height. While walking outside, the patient slipped on a patch of ice and fell. She had immediate abdominal and lower back pain. At the outside hospital, the patient was found to be tachycardic and hypotensive with a systolic blood pressure of 80/40 millimeters of mercury (mm Hg). Her right upper quadrant view on eFAST was positive for free abdominal fluid. Her initial hemoglobin and hematocrit were 8.3 grams per deciliter (g/dL) and 32.7%, respectively. She was started on two units of packed red blood cells, 1 g of tranexamic acid, and 1 liter (L) of intravenous (IV) fluids and was transferred for further trauma evaluation. On arrival, the patient denied recent illnesses or influenza-like symptoms. She reported possible dizziness or lightheadedness before the fall but was uncertain whether any symptoms truly preceded it. She denied abdominal pain or back pain prior to the fall. The patient denied current chest pain or shortness of breath.

Her initial vitals showed a heart rate of 139 beats per minute (min), blood pressure of 113/65 mm Hg, respiratory rate of 25 breaths per min, and oxygen saturation of 100% on four L/min of oxygen via nasal canula. Notably, all subsequent systolic blood pressures were in the 60s–70s. She was alert, oriented, answering all questions appropriately, and following commands with a Glasgow Coma Scale of 15. On exam, she was in significant pain and appeared pale. Her lungs were clear to auscultation with equal breath sounds bilaterally. She was tachycardic with a regular heart rhythm. Her abdomen was soft and diffusely tender to palpation with voluntary guarding. She did not have midline spinal tenderness or paraspinal tenderness. She had palpable radial and dorsalis pedis pulses bilaterally. Notable laboratory results are shown in the Table. A basic metabolic panel was within normal limits (not shown). Because point-of-care testing was not available, we had no lab results e during initial management.

**Figure 1:**
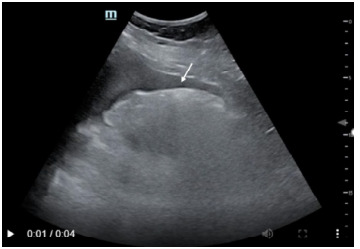
Right upper quadrant view from extended focused assessment with sonography in trauma with white arrow demonstrating free fluid.

Her eFAST exam was remarkable for free fluid in the right upper quadrant (Figure 1), left upper quadrant, and suprapubic views. There was no pericardial effusion on subxiphoid view. We did not obtain additional cardiac views or views of the inferior vena cava on initial presentation. Right heart strain was not evaluated for on initial eFAST exam.

**Figure 2:**
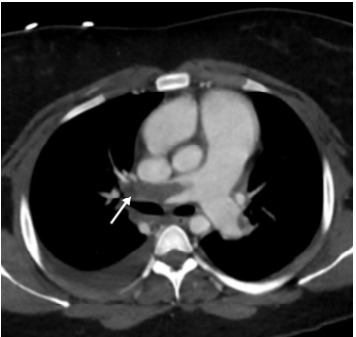
Near-complete filling defect of the right main pulmonary artery consistent with pulmonary embolism seen on computed tomography of the chest and denoted by white arrow.

**Figure 3:**
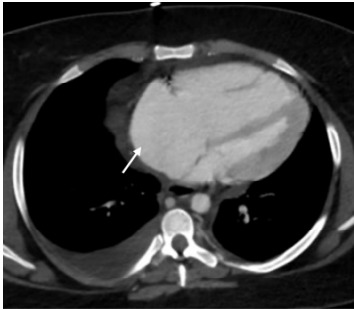
Enlargement of the right ventricle and right atrium (white arrow) seen on computed tomography.

We established additional IV access and an arterial line for accurate blood pressure monitoring. As repeat lab results were not available, and in the absence of other causes of the the patient’s hemodynamic stability, we initiated massive transfusion protocol for presumed hemorrhagic shock. Given relative stability and appropriate mentation after initial resuscitative measures, in conjunction with our trauma surgery team, we opted to pursue cross-sectional imaging with computed tomography (CT) prior to mobilizing the patient to the operating room for exploratory laparotomy. Her CT was remarkable for bilateral PE with a large thrombus in the right main pulmonary artery with enlargement of the right atrium and ventricle demonstrating evidence of right heart strain (Figures 2 and 3).

She also had a non-occlusive thrombus within the inferior vena cava. The CT also demonstrated a large volume of free fluid within the pelvis, characterized as slightly complex in the posterior cul-de-sac and adjacent to the right ovary, although predominantly hypoattenuating. A 1.4 centimeter (cm) corpus luteal cyst was noted within the right ovary. The liver exhibited a nutmeg appearance with associated mild-to-moderate periportal edema and moderate ascites. No evidence of traumatic injury was identified.

*CPC-EM Capsule*

What do we already know about this clinical entity?

*Extended Focused Assessment with Sonography for Trauma (EFAST) examinations are a vital source of information in unstable trauma patients and may help guide further treatment and interventions*.

What makes this presentation of disease reportable?

*Unstable trauma patient with positive intraabdominal free fluid found to have ruptured ovarian cyst/ascites and massive pulmonary embolism (PE)*.

What is the major learning point?

*EFAST examinations have high specificity, but there are important false positive causes that can lead to additional interventions which providers should be aware of*.

How might this improve emergency medicine practice?

*Emergency physicians should be aware of limitations and false positive causes of intraabdominal free fluid in EFAST exams*.

Given uncertainty about the origins of the abdominal fluid, we deferred administering tissue plasminogen activator. A heparin drip was started, and the patient was admitted to the intensive care unit in stable condition. Shortly after admission she was taken emergently to the catheterization lab for aspiration thrombectomy with interventional radiology. The procedure was unsuccessful, and catheter-directed thrombolytic therapy was performed. Her inpatient course was complicated by a right groin hematoma and acute blood loss anemia requiring transfusion, as well as significantly elevated pulmonary artery pressures. She was discharged after an 18-day hospital stay on apixaban and sildenafil for chronic thromboembolic pulmonary hypertension. Extensive outpatient workup did not identify a cause of her PE.

## DISCUSSION

Prompt identification of the etiology of shock is essential in preventing its sequelae including multiorgan failure and death. Even in cases where there appears to be a clear cause of shock, maintaining a high index of suspicion for mixed shock or alternative causes can prevent premature closure. The incidence of PE in ED patients under the age of 21 is approximately 2.1 per 100,000 visits,^8^ and it is even lower in patients who do not have any discernable risk factors, making our patient’s ultimate diagnosis an unusual and unexpected cause of shock.

Although blunt trauma is a common cause of hemorrhagic shock,^9^ falls from standing height rarely result in intra-abdominal injury significant enough to require procedural intervention in this patient age.^10^ Otherwise healthy young adults who sustain injuries from falling from standing height most commonly experience orthopedic injuries^11^ and largely do not require admission.^12^ Women often attribute falls to external factors and may downplay symptoms suggestive of syncope.^13^ In the case of our patient, she attributed her fall to slipping on ice and only later posited that she may have felt dizzy preceding her fall.

The patient’s initial presentation was as a transfer from another hospital, and a prehospital trauma alert was placed prior to her arrival. Information received pre-arrival led the emergency department (ED) and trauma teams to frame her case as one of hemorrhagic shock secondary to a fall. In her case, however, the patient’s positive eFAST findings were likely instead related to rapid accumulation of ascites and periportal edema consistent with acute right heart failure and hepatic congestion^14^ and were likely exacerbated by the aggressive administration of IV fluids and blood products both in the prehospital setting and upon arrival.

Although a corpus luteal cyst with a small amount of complex fluid in the posterior cul-de-sac was identified, it is unlikely that a ruptured hemorrhagic cyst alone accounted for the grossly positive eFAST exam. One study reported an average volume of 66 mL for hemorrhagic ovarian cysts,^15^ typically insufficient to result in such significant free fluid. Interestingly, a framing effect may have led to early diagnostic closure, particularly when her eFAST exam provided positive results. Given the patient’s initial insistence that the fall was not preceded by prodromal symptoms, and the absence of reported right heart strain on the initial eFAST performed by the trauma team, early testing for syncope-related causes was not pursued despite the patient requiring 4 L/min oxygen via nasal cannula to maintain adequate oxygenation.

Often intertwined with the framing effect, anchoring bias involves an over-reliance on initial information during diagnostic reasoning and may contribute to premature closure,^16^ such as attributing hypotension solely to hemorrhagic shock from trauma without fully considering alternative causes. A recent study found that patients presenting with shortness of breath and triaged with congestive heart failure (CHF) were less likely to be evaluated for PE compared to those without a specific diagnosis mentioned, despite similar PE rates. This may reflect anchoring or framing bias related to known CHF history.^16^ In our patient’s case, prehospital information suggesting a traumatic mechanism may have misdirected her initial diagnostic workup and management. The eFAST was approached primarily as an assessment for acute traumatic injuries – especially in light of her report of trauma and positive intra-abdominal fluid seen on outside hospital ultrasound and full cardiac evaluation was not performed on initial examination. This narrow focus, coupled with premature closure on the diagnosis of hemorrhagic shock both at the outside hospital and upon arrival to our ED, may have led to rapid volume expansion that further impaired right ventricular function and reduced cardiac output.^14^,^17^

## CONCLUSION

The extended focused assessment with sonography in trauma is a critical aspect of the initial trauma evaluation in unstable patients who present to the ED. However, in the case of a positive eFAST exam in a patient who falls from standing height, contextualizing risks of intra-abdominal trauma is critical to interpreting the eFAST. Indeed, abdominal trauma requiring intervention in individuals who fall from standing height is extremely uncommon.^10^ It is essential to maintain a high index of suspicion for alternative causes of both the fall and the shock in unstable patients presenting after a fall from standing height, while also critically reflecting on potential biases that may influence the interpretation of the patient’s presentation.

## Figures and Tables

**Table:** Notable laboratory results on initial presentation to the emergency department.

Lab Test	Result	Reference Range
**Complete Blood Count**		
WBC	8.0 × 10^3^ cells/μL	4.0–10.0 × 10^3^ cells/μL
Hgb	18.6 g/dL	11.2–15.7 g/dL
Hct	61.7%	34%–45%
Plt	50 × 10^3^/μL	150–400 × 10^3^/μL
**Coagulation Panel**		
PT	21.4 s	9.4–12.5 s
PTT	39.6 s	25.0–36.5 s
INR	1.9	0.9–1.1
**Venous Blood Gas**		
pH	7.19	7.35–7.45
pO_2_	71 mm Hg	85–105 mm Hg
pCO_2_	38 mm Hg	35–45 mm Hg
Lactate	5.0 mmol/L	0.5–2.0 mmol/L
**Cardiac Biomarkers** *		
Troponin	0.22 ng/mL	0–0.01 ng/mL
proBNP	197 pg/mL	0–178 pg/mL

* Cardiac biomarkers were not included in the initial order set.

Troponin and proBNP were sent after computed tomography demonstrated pulmonary embolism with associated right heart strain.

Abbreviations*: ED*, emergency department; *WBC*, white blood cell*s*; *μL*, microliters; *Hgb*, hemoglobin; *dL*, deciliters; *Hct*, hematocrit; *Pl*t, platelets; *PT*, prothrombin time; *PTT*, partial thromboplastin time; s, seconds; *INR*, international normalized ratio; *pO_2_*, partial pressure of oxygen; *pCO_2_*, partial pressure of carbon dioxide; *ng*, nanograms; *pg*, picograms; *proBNP*, pro B-type natriuretic peptide.
